# Expression of Glutamine Transporter Slc38a3 (SNAT3) During Acidosis is Mediated by a Different Mechanism than Tissue-Specific Expression

**DOI:** 10.1159/000358722

**Published:** 2014-05-16

**Authors:** Sarojini Balkrishna, Angelika Bröer, Scott M. Welford, Maria Hatzoglou, Stefan Bröer

**Affiliations:** a Research School of Biology, Australian National University, Canberra, ACT, Australia; b Department of Radiation Oncology, School of Medicine, Case Western Reserve University, Cleveland, OH, USA; c Case Western Reserve University, School of Medicine, Wood Research Tower 600 Cleveland, OH, USA

**Keywords:** Amino acid transport, SN1, Promoter methylation, Gene regulation

## Abstract

**Background:**

Despite homeostatic pH regulation, systemic and cellular pH changes take place and strongly influence metabolic processes. Transcription of the glutamine transporter SNAT3 (Slc38a3) for instance is highly up-regulated in the kidney during metabolic acidosis to provide glutamine for ammonia production.

**Methods:**

Slc38a3 promoter activity and messenger RNA stability were measured in cultured cells in response to different extracellular pH values.

**Results:**

Up-regulation of SNAT3 mRNA was mediated both by the stabilization of its mRNA and by the up-regulation of gene transcription. Stabilisation of the mRNA involved a pH-response element, while enhanced transcription made use of a second pH-sensitive Sp1 binding site in addition to a constitutive Sp1 binding site. Transcriptional regulation dominated the early response to acidosis, while mRNA stability was more important for chronic adaptation. Tissue-specific expression of SNAT3, by contrast, appeared to be controlled by promoter methylation and histone modifications.

**Conclusions:**

Regulation of SNAT3 gene expression by extracellular pH involves post-transcriptional and transcriptional mechanisms, the latter being distinct from the mechanisms that control the tissue-specific expression of the gene.

## Introduction

The extracellular and intracellular pH is held within narrow limits in most tissues and cells. Despite this homeostatic control, the proton concentration can change 2–3 fold in a number of conditions [1]. For instance, the extracellular pH can decrease below 7.0 due to metabolic acidosis. This can occur as a result of excessive production of organic acids such as lactate, hydroxybutyrate and acetoacetate during starvation and uncontrolled diabetes [2]. Cancer cells generate local extracellular acidosis, while keeping the intracellular pH at pH 7.5, about 0.3 units more alkaline than normal cells [1]. Many enzymes and transporters are exquisitely pH sensitive in the physiological range. The rate of glycolysis for example is determined in part by pH regulation of phosphofructokinase-1 [3]. Kidney metabolism is also highly responsive to changes of extracellular pH. During chronic acidosis, the kidney extracts glutamine from the circulation, converting it to glutamate and subsequently α-ketoglutarate [4]. These reactions release ammonia and are carried out by glutaminase and glutamate dehydrogenase, respectively [5]. Ammonia attracts a H^+^ to form ammonium ions, which are then released with the urine. At normal pH, the kidney extracts little glutamine from the circulation, while during chronic metabolic acidosis net extraction can reach 30% [6]. Glutamine is extracted from the circulation through the basolateral membrane via the glutamine transporter SNAT3 (gene name Slc38a3) [7–9]. Under physiological conditions expression of SNAT3 is restricted to the outer medullary S3 segment of the proximal tubule. During metabolic acidosis caused by treatment of rats with ammonium chloride, SNAT3 expression increases in both, the kidney cortex and the outer medullary stripe [8]. A corresponding increase of Na^+^-dependent glutamine transport is observed in basolateral membrane vesicles derived from cortex, whereas Na^+^-independent transport remained constant. The increased expression of SNAT3 in the proximal tubule also coincides with that of the enzymes involved in the metabolism of glutamine and gluconeogenesis, such as glutaminase, phosphenolpyruvate carboxykinase (PEPCK), fructose-1,6-bisphosphatase [FBPase] and glucose-6-phosphatase (G6Pase) [10]. The conversion of α-ketoglutarate into glucose generates two HCO_3_^−^, which combine with protons to from carbonic acid [10]. This also compensates acidosis after breakdown of carbonic acid into H_2_O and CO_2_ and release through the lungs. Notably, carbonic acid anhydrase interacts with SNAT3, providing a link between removal of HCO_3_^−^ and glutamine transport [11].

LLC-PK1-FBPase^+^ cells have been used as a model system to study pH-regulation in the kidney [12]. The cells have features of proximal tubular cells, but more importantly, show increased glutamine metabolism and gluconeogenesis in response to acidotic media (customarily a change from pH 7.4 to 6.9) [13]. Thus, these cells undergo a normal physiological response to acidosis through the up-regulation of both, gluconeogenesis and glutamine metabolism. The up-regulation of glutaminase is mediated by stabilization of its mRNA through an AU-rich element (ARE) in the 3′UTR, called the pH-response element (pH-RE) [14]. This element is also conserved in the 3′UTR of mouse SNAT3 mRNA (UUUUAUAAAUAUUAUA) and has been shown to interact with an unidentified RNA-binding protein [8]. It has been proposed that ζ-crystalline, an NADPH-dependent redox enzyme can bind to the pH-RE, preventing degradation by RNases [15]. Glutaminase mRNA can also bind to AU-binding proteins, AUF1 and HuR [16], which affect mRNA stability. Initial increases in *Pepck* mRNA levels occur within one hour of acidosis (acute) due to increased rates of transcription through the activation of the kinase p38 and Activating Transcription Factor-2 (ATF-2) signalling pathway. However, chronic acidosis (more than 7h) sustains the *Pepck* mRNA by increasing its stability [14]. *In vitro* studies in LLC-PK1F^+^ cells showed that under chronic acidosis, *Pepck* is stabilized due to the concurrent binding of HuR and AUF1 to the ARE in its 3′UTR [17].

Given the common mechanisms that govern the up-regulation of enzymes involved in the metabolism of glutamine during acidosis, it is tempting to speculate that SNAT3 would also be regulated in a similar manner. A pH-RE has been identified in the SNAT3 3′UTR and gel-shift assays suggested protein binding to this element [8]. Whether the presence of this element leads to increased stability of SNAT3 mRNA during acidosis is unknown. Here, we show that SNAT3 3′UTR contains a functional pH-RE, which stabilizes the mRNA during long-term changes of extracellular pH. Furthermore, the SNAT3 promoter is also transcriptionally up-regulated in response to changes to the extracellular pH.

## Materials and Methods

### Plasmids and constructs

Genomic DNA was extracted from mouse liver as per the instructions of the DNeasy Blood and Tissue kit (Qiagen). This DNA was used as a template to amplify the *Slc38a3* promoter region ranging from −*1933 to +49,* relative to the transcriptional start site. The amplified product was digested with KpnI and XhoI and ligated into a likewise cut pGL4.12 firefly luciferase reporter vector (Promega). This generated a construct where the *Slc38a3* promoter region ranging from −*1841 to +49* was inserted in front of the luciferase gene (−1841-luc). Successful ligations were rapidly screened and sequenced at the Biomolecular Resource Facility at the Australian National University before use. Deletions of the −1841-luc construct were created by overlap extension PCR using the QuickChange II Site directed mutagenesis kit (Stratagene). All primers used for the creation of these constructs are outlined in Table 1. The PCR products were treated with Polynucleotide kinase (New England Biolabs) and the phosphorylated products ligated using the Quick Ligation Kit (New England Biolabs). Ligated products were rapidly screened and sequenced. Site-directed mutagenesis was performed as per the instructions of the QuickChange II Site directed mutagenesis kit (Stratagene). All mutants were sequenced at the Biomolecular Resource Facility at the Australian National University before use. The primers used for this purpose are outlined in Table 1.

### Cell culture and acidosis

The following cells were grown and maintained at 37°C in a cell incubator with 5% CO_2_ and 95% air in the media described: HepG2: DMEM/Ham’s F12/10%FCS/2mM glutamine; FRT cells: DMEM/F12/5% FCS/2mM glutamine; HEK293: RPMI1640/5% FCS/2mM glutamine; HeLa: DMEM/10%FCS/2mM glutamine; Sp^1−/−^: α-MEM/5%FCS/1ng/ml basic fibroblast growth factor/4μg/ml insulin/1mM glutamine; MEF: DMEM/10%FCS/2mM glutamine.

LLC-PK1F^+^ cells were cultured as described before [18]. Acidosis was induced by decreasing the amount of bicarbonate in the media for a 7.5% CO_2_ cell incubator at 37°C. 2.37g/L of NaHCO_3_^−^ was used to obtain a final media pH of 7.4 and 0.75g/L of NaHCO_3_^−^ was used to obtain a final media pH of 6.9. The amount of ammonia excreted by cells after treatment with this media was measured using the Ammonia Assay Kit (Sigma-Aldrich). The amount of ammonia was normalized to protein quantities in each dish as determined with the Bradford Reagent (Pierce).

### Extraction of mouse primary renal cortical tubule cells

Extraction of cortical tubule cells were performed as described before [19]. The protocol was approved by the institutional review board of Case Western Reserve University. The kidneys from a 2–3 week old C57BL6J mouse were extracted. The capsule was removed and the kidney was cut longitudinally with a sterile blade. The visible cortex was excised and minced in Hanks Buffered Saline Solution (HBSS), pH 7.4. The minced kidneys were placed in a collagenase solution (1mg/ml Collagenase IV and 1mg/ml trypsin inhibitor made in HBSS, pH 7.4) and incubated at 37°C for 15 minutes. The cells were dissociated by mixing and incubated for a further 15 minutes at 37°C. The collagenase reaction was stopped by adding 1.5ml FCS per 10ml of collagenase solution and cells were isolated by centrifugation for 5 minutes at 500rpm at 4°C. The cells were resuspended in HBSS (pH 7.4) and centrifuged for 5 minutes at 500rpm at 4°C. The pellet was resuspended in media (DMEM/F12-high glucose, 5mg/ml transferrin, 5μg/ml insulin, 0.01μg/ml hydrocortisone) and distributed into cell culture dishes. The distribution of cells from one mouse was used to create two 100mm or six 60mm cell culture dishes. Epithelial cells attached to the dishes overnight and the media was changed every 24 hours to allow cell proliferation.

### Reverse Transcription-Polymerase Chain reaction (RT-PCR)

RNA Extractions from cells or tissues were performed as per the instructions of the Nucleospin RNA II kit (Macherey Nagel). 1μg RNA was reverse transcribed to cDNA as per the instructions accompanying the Superscript II Reverse Transcriptase enzyme (Invitrogen). The resulting cDNA was used for qualitative or quantitative PCR analysis. For qualitative PCR analysis, the cDNA was purified using a PCR purification kit (Invitrogen). Of the purified cDNA, 1–2μl was subjected to PCR with Taq polymerase (Invitrogen) or Pfu polymerase (Promega). In each case, PCR conditions were followed according to the kit instructions. PCR products were then analysed by agarose gel electrophoresis (1–2%) The primers used for this purpose are listed in Table 1.

Quantitative Real-Time PCR was performed on the 7900HT Real-Time System (Applied Biosystems). The relative expression of target genes was analysed by the SDS 7900 Software. Primers used for this purpose are listed in Table 1. The dissociation curves and primer efficiencies were monitored using the SDS 7900 Software.

### Dual-Iuciferase Assay

The Dual-luciferase Assay was performed as per the instructions of the Dual-Luciferase® Reporter Assay System (Promega). Experiments were performed on 24-well plates. For each condition, 500ng of the Firefly constructs and 5ng of the Renilla vector were transfected using Lipofectamine LTX (Invitrogen). The total amount of DNA transfected per condition was kept constant by topping up with the pCDNA3.1(+) vector (Invitrogen). Reactions were scaled down so that 20μl LARII buffer, 20μl lysed sample, and 20μl Stop and Glow buffer (Promega) were used per reaction. Luminescence was measured on a TD20/20 Luminometer (Turner Designs) with the Dual Luciferase function. Data was represented as the ratio of luminescence emitted by the firefly vector to luminescence emitted by the Renilla vector.

### Chromatin Immunoprecipitation

Chromatin Immunoprecipitation (ChIP) was performed as per the instructions of the SimpleChIP Enzymatic Chromatin IP Kit (Cell Signaling). For HepG2 cells, no deviations were made to the prescribed protocol. For each immunoprecipitation from mouse tissue, 25mg of fresh or snap-frozen mouse tissue was minced with a scalpel and placed in 1ml PBS containing a protease inhibitor cocktail (cOmplete EDTA-free, Roche). The proteins and DNA were then cross-linked in 1.5% formaldehyde for 20 minutes at room temperature. The reaction was stopped by the addition of glycine (final 1x from a 10x stock provided with the kit) for 5 minutes. The tissue was centrifuged for 5 minutes at 1500 rpm at 4°C and then washed once with 1ml PBS containing protease inhibitors. After disaggregating the tissue with a potter homogenizer using 20–25 strokes, the cell suspension was centrifuged for 5 minutes at 4°C at 1500 rpm. The supernatant was immediately removed from the cells and nuclei were prepared as per the instructions of the kit. Chromatin was digested with 0.5μl Micrococcal Nuclease for 45 minutes. This was followed by 2 minutes of sonication (Misonix S4000 sonicator) at 80% amplitude with a 15 second pulse on and 15 second pulse off time. This was followed by 2 minutes of sonication at 100% amplitude with a 15 second pulse on and 15 second pulse off time. Lysates were clarified by centrifugation at 10000 rpm for 10 minutes at4°C. The supernatant containing the cross-linked chromatin preparation was incubated overnight in 1–5 μg of anti-Histone H3 (positive control), IgG (negative control), Sp1 (Abcam – ab13370, USA) or RNA Polymerase II (Santacruz SC-899) antibodies. DNA was purified as per the instructions of the kit and analysed using PCR. The primers used for PCR from ChIP samples are outlined in Table 1.

### Bisulfite Genome Sequencing

Genomic DNA from mouse liver and intestine was isolated using the DNeasy Blood and Tissue kit (Qiagen). Genomic DNA (500ng) was treated with bisulfite as per the instructions of the EpiTect Bisulfite conversion kit (Qiagen). The bisulfite conversion reaction was performed in a single tube in a thermal cycler with the following conditions: 99°C – 5 min, 60°C – 25 min, 99°C – 5min, 60°C – 85min, 99°C – 5min, 60°C – 175min. Bisulfite converted DNA was purified as per the instructions of the kit, and 20μl of the eluted DNA was subjected to a second round of bisulfite conversion to ensure maximum conversion of cytosine residues. Purified DNA was subjected to nested PCR by *Taq* Polymerase using the primers listed in Table 1. Nested PCR fragments were analysed on an agarose gel, after which the PCR product was purified and cloned in to the pCR TOPO XL vector as per the instructions provided in the kit (Invitrogen). 10–15 clones were sequenced and analysed using the BioQ Analyzer software [20]. Conversion efficiencies of 99–100% were obtained for all reactions. Statistical significance was obtained by performing a chi-square and Fisher’s test on the Odd’s ratios for methylation patterns comparing the liver and intestine.

### mRNA half-life analysis

Primary cells or cell lines were grown to 70% confluency on 60mm dishes. Cells were treated with Actinomycin D (10μg/ml, Sigma, USA) for the indicated time points, and RNA was extracted using TriZol (Invitrogen, USA). The quality of the extracted RNA was validated using gel electrophoresis. cDNA was prepared with the Superscript III kit (Invitrogen, USA) and quantitative real-time PCR was performed with the Power SYBR Green PCR Master mix and samples were analysed on a StepOne Plus Real Time PCR System (Applied Biosystems, USA). The primers used for this purpose are outlined in Table 1. mRNA levels were normalized to 18 S or GAPDH and expressed as percentages of the levels before the addition of Actinomycin D [21].

### Bioinformatic analyses

The UCSC Genome Browser is accessible at http://genome.ucsc.edu/index.html [22] and was used to access the genomic sequences of *Slc38a3.* The ENCODE data sets were accessed through the same portal. Transcription factor searches were performed on the MatInspector function of Genomatix, accessible at http://www.genomatix.de/online_help/help_matinspector/matinspector_help.html#references [23]. CpG island searches were performed on the CpG Cluster on UCSC Browser and the USC Norris CpG Island Searcher accessible at http://www.uscnorris.com/cpgislands2/cpg.aspx [24].

### Statistics

All statistical analyses were performed on the GraphPad InStat program.

## Results

### The SNAT3 mRNA is stabilized during acidosis

**Fig. 1:**
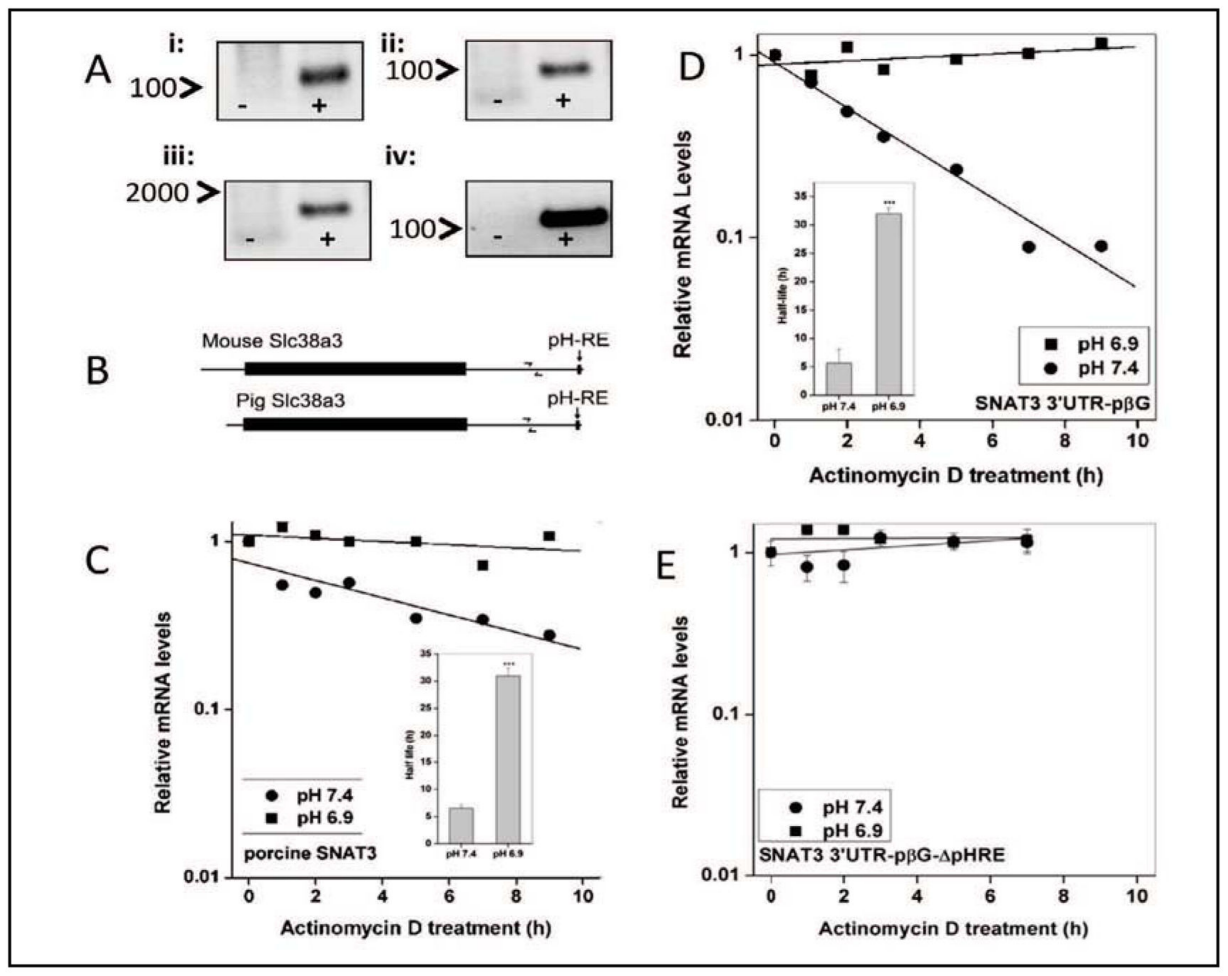
Stability of SNAT3 mRNA during acidosis. A) PCR without (−) or with added reverse transcriptase reactions (+) of LLC-PK1F^+^ cells with primers spanning the 5′ end of the gene (position 392–500 for pig Slc38a3 accession number XM_005669590.1) (i), 3′UTR (position 2012–2113) (ii), (iii) porcine SNAT3 mRNA (392-2113); and the housekeeping ribosomal 18S rRNA (iv). B) Scheme of mouse and pig SNAT3 mRNAs, depicting primers to detect the mRNA in the 3′UTR and the position of the pH-RE. C) LLC-PK1F^+^ cells were treated with normal (pH 7.4) or acidic (pH 6.9) medium for 12h. Subsequently, cells were treated with Actinomycin D to stop transcription, and mRNA was extracted at the indicated times. Porcine SNAT3 mRNA was quantified relative to the ribosomal 18S rRNA. At pH 7.4, porcine SNAT3 mRNA had *t*_1/2_=6h and was stabilized (*t*_1/2_>10h) after acidotic treatment. Each point indicates the mean±s.d. of triplicates of relative mRNA levels. Summary statistics of two independent experiments is shown in the insert. D) LLC-PK1F^+^ cells were transfected with mSNAT3-3′UTR-pβG. Cells were treated with normal (pH 7.4) or acidic (pH 6.9) medium for 12h followed by Actinomycin D treatment for the indicated times. Mouse SNAT3 mRNA was quantified relative to the ribosomal 18S rRNA. At pH 7.4, SNAT3-3′UTR-pβG mRNA had *t*_1/2_=2.5h and was stabilized (*t*_1/2_>10h) after acidotic treatment. Each point indicates the mean±s.d of triplicates of relative mRNA levels. Summary statistics of three independent experiments is shown in the insert. E) LLC-PK1F^+^ cells were transfected with SNAT3-3′UTR-pβG containing a deletion of the AU-repeat element. Cells were treated with normal (pH 7.4) or acidic (pH 6.9) medium for 12h followed by Actinomycin D treatment for the indicated times. Mouse SNAT3 mRNA was quantified relative to the ribosomal 18S rRNA. The resulting mRNA was very stable (*t*_1/2_>10h) at normal and acidotic conditions. Each point indicates the mean±s.d of triplicates of relative mRNA levels. Each bar represents the mean±s.d of *t*_1/2_ of either porcine SNAT3 mRNA (C) or mSNAT3-3′UTR-pβG mRNA at pH 7.4 and 6.9 (D). *** indicates p<0.001 for the student’s *t* test comparing the mean *t*_1/2_ at pH 7.4 to pH 6.9

LLC-PK1-FBPase^+^ cells have been used frequently to study the response of renal tubular cells to acidosis. The cell line expresses endogenous porcine SNAT3 (Fig. 1A), which has the same pH-RE at the 3′ end of its mRNA as mouse SNAT3 mRNA (Fig. 1B). At pH 7.4 the half-life of porcine SNAT3 mRNA in LLC-PK1-FBPase^+^ cells was 6h. Incubation of these cells in acidic media (pH 6.9) resulted in increased mRNA stability with a t_1/2_ > 30h (Fig. 1B, C inset shows summary statistics). To see whether the 3′UTR of mouse SNAT3 contained an element that would destabilize its mRNA at neutral pH, we cloned the complete 770bp mouse SNAT3 3′UTR (Fig. 1B) into the pβ-globin vector (pβG), which has previously been used to study the effects of acidosis on glutaminase mRNA stability. The unmodified β-globin mRNA is very stable (*t*_1/2_ > 30h) in LLC-PKl-FBPase^+^ cells [18]. Insertion of elements from the 3′UTR of other genes allows studying their influence on mRNA stability. Insertion of the mSNAT3-3′UTR into the pβG mRNA destabilized the otherwise stable β-globin mRNA at pH 7.4 (*t*_1/2_ = 5h, Fig. 1D). Acidotic treatment (12h at pH 6.9) of these cells caused a stabilization of the mSNAT3-3′UTR-pβG construct (*t*_1/2_ > 30h, Fig 1D, inset shows summary statistics). Deletion of the putative pH-RE in the mSNAT3-3′UTR-pβG construct resulted in a general stabilisation of the mSNAT3-3′UTR-pβG RNA (t_1/2_ > 30h, Fig. 1E). These results indicate that the conserved AU-rich region in the SNAT3 3′UTR contains elements that destabilize a heterologous mRNA, and that masking of this element is responsible for mRNA stabilization during acidosis.

**Fig. 2:**
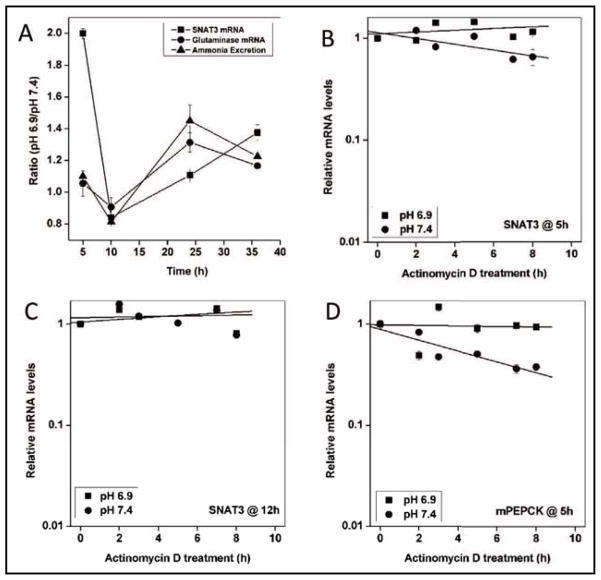
Acidosis and mRNA stability in mouse primary renal cortical tubules. Primary cultures of mouse renal cortical tubules were incubated in normal (pH 7.4) or acidotic (pH 6.9) medium for the indicated time points. A) The amount of ammonia excreted and relative levels of SNAT3 and mitochondrial glutaminase mRNA was determined at these time points. Each data point indicates the mean±s.d. of the fold change values obtained from three separate measurements. One of two independent repeats is depicted. Ammonia excretion varied from 1.2–1.8 μg/ml*mg protein, mouse glutaminase mRNA levels varied from 105–162% control and mouse Slc38a3 mRNA levels varied from 14–51% control in in the primary cultures. After 5h (B) or 12h (C, D) cells were treated with Actinomycin D for the indicated time points. Mouse SNAT3 mRNA (B, C) or Pepck mRNA (D) was quantified relative to the ribosomal 18S rRNA. For each condition, SNAT3 mRNA had t_1/2_>10h. For *Pepck*, t_1/2_ at pH 7.4 = 5.5h and, t_1/2_ at pH 6.9>10h. Each point indicates the mean±s.d of triplicates mRNA level measurements.

To see whether a similar effect is also observed in a system more closely related to the kidney cortex, we used primary cultures of kidney cortex tubular cells. When exposed to acidotic conditions, the cells produced ammonia thus showing an increased use of glutamine. The increase of ammonia levels (given as ratio of ammonia production over control) are similar to those observed in previous studies using LLC-PK1-FBPase^+^ cells [25, 26]. Acidosis-induced ammonia excretion varies from ~1.2–2 fold, which compares well to a ~2-fold increase in glutaminase mRNA levels reported previously [27–29]. The SNAT3 mRNA levels showed a biphasic response (Fig. 2A). There was a fast two-fold up-regulation of SNAT3 mRNA in the first 5h under acidotic conditions. This initial peak ceased at 10h and was followed by a slow increase of SNAT3 mRNA over 36h. Glutaminase mRNA showed a slight 1.2-fold increase compared to pH 7.4. Ammonia excretion closely followed glutaminase expression. Thus apart from the initial increase of SNAT3 mRNA, primary cultures showed only a limited adaptation to acidic pH. Accordingly a small stabilisation of SNAT3 mRNA was observed after 5h at acidic pH, whereas at later time points the mRNA was generally stable (*t*_1/2_ >20h, Fig. 2B, C). Stabilisation from *t*_1/2_ of 5.5h (pH 7.4) to *t*_1/2_>10h (pH 6.9) was also observed for the Pepck mRNA during chronic acidosis (Fig. 2D). These values are comparable to previously obtained results in LLCPK1-FBPase^+^ cells (*t*_1/2_ = ~3h at pH 7.4) [29]. Overall, the experiments suggest that the SNAT3 mRNA is already expressed at significant levels at pH 7.4 in primary cultures derived from renal cortical tubules and that its mRNA is relatively stable.

### Structure of the SNAT3 promoter

**Fig. 3:**
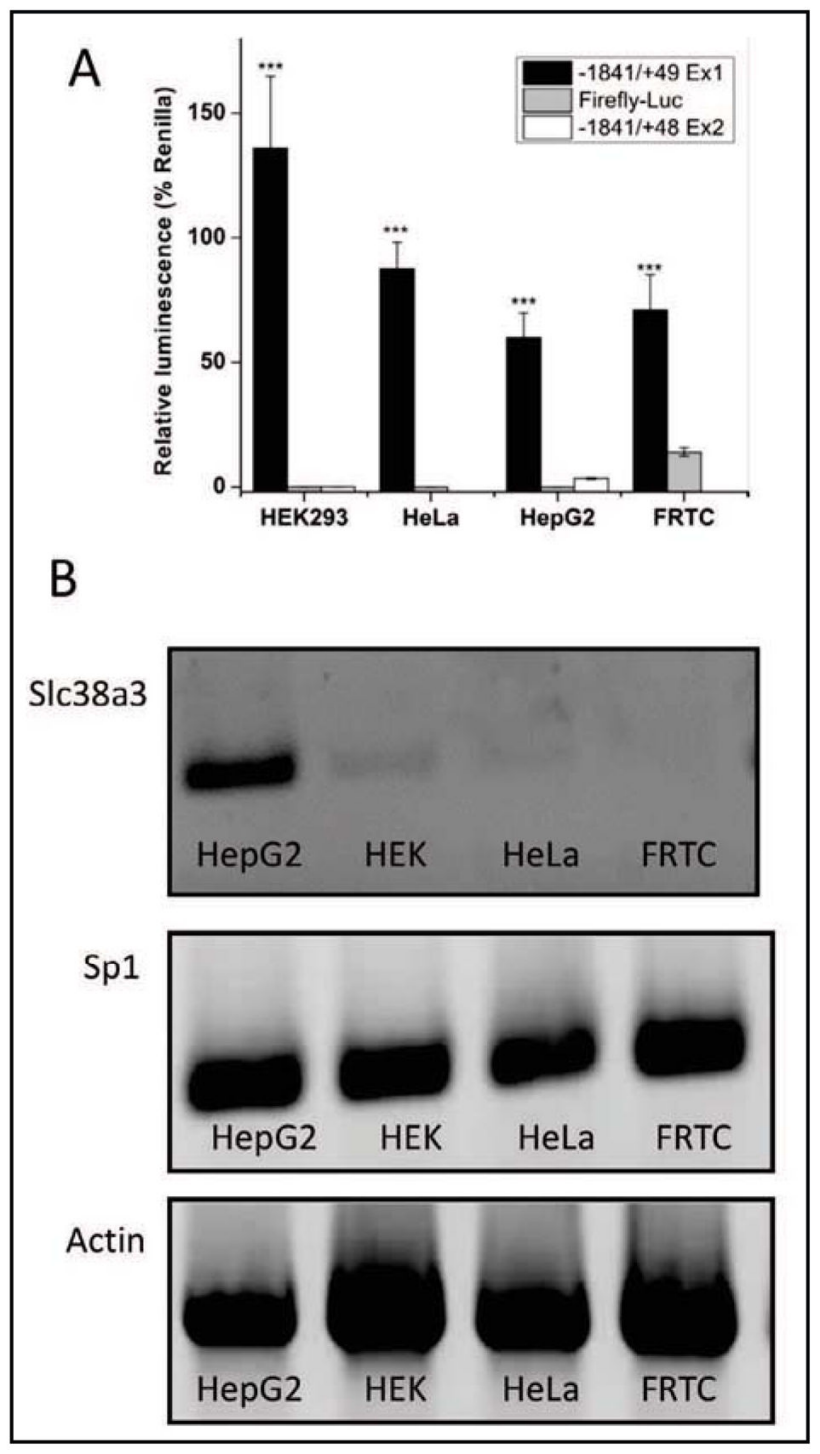
*Slc38a3* promoter activity in different cell lines. A) The relative luminescence of the −1841/+49-luc Ex1 construct (upstream Exon 1) was measured in HEK293, HeLa, HepG2 and FRTC cells, the relative luminescence of (−1841/+48-luc Ex2 construct (upstream Exon 2) was measured in HEK293 and HepG2 cells, only. Each bar represents the mean±s.d. of three dishes. One of three independent repeats is depicted. *** corresponds to *p*<0.0001 for the student’s *t* test comparing −1841/+49-luc activity to the corresponding promoter-less firefly-luc control. Transcriptional activity of the promoter was independent of *Slc38a3* expression in the cell lines. B) RT-PCR of human SNAT3 (position 1184–1599 for hSlc38a3 accession number NM_006841) and *Sp1* was performed from HepG2, HEK293, HeLa and FRTC cells. The housekeeping gene actin was used as a control.

**Fig. 4:**
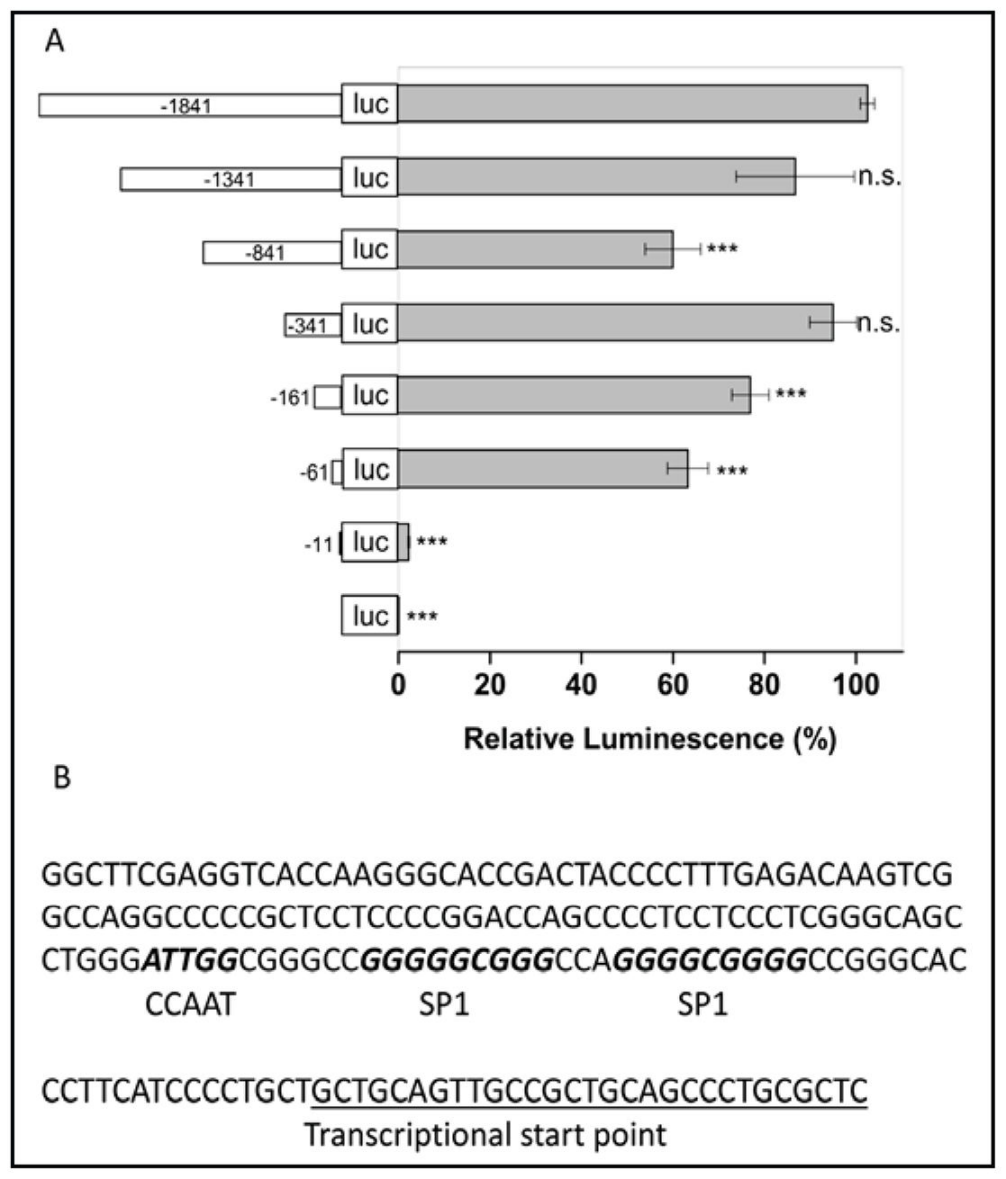
Activity of *Slc38a3* promoter deletion constructs. A) Deletions of the −1841/+49-luc *Slc38a3* promoter construct were transfected into HEK293 cells. Every deletion also contained the *Slc38a3* +49 region upstream of the luciferase gene. The relative luminescence of these constructs was measured using the Dual-Luciferase assay. Each bar represents the mean±s.d. of the luminescence from three dishes. One of three independent experiments is depicted. *** indicates *p*<0.0001 and n.s. indicates no significance for the student’s *t* test comparing the deletion constructs to the control (−1841/+49-luc). B) Using the MatInspector and UCSC Genome Browser, two Sp1 and one CCAAT enhancer (bold italics) were detected in the region 50 bp upstream of the transcription start-site.

**Fig. 5:**
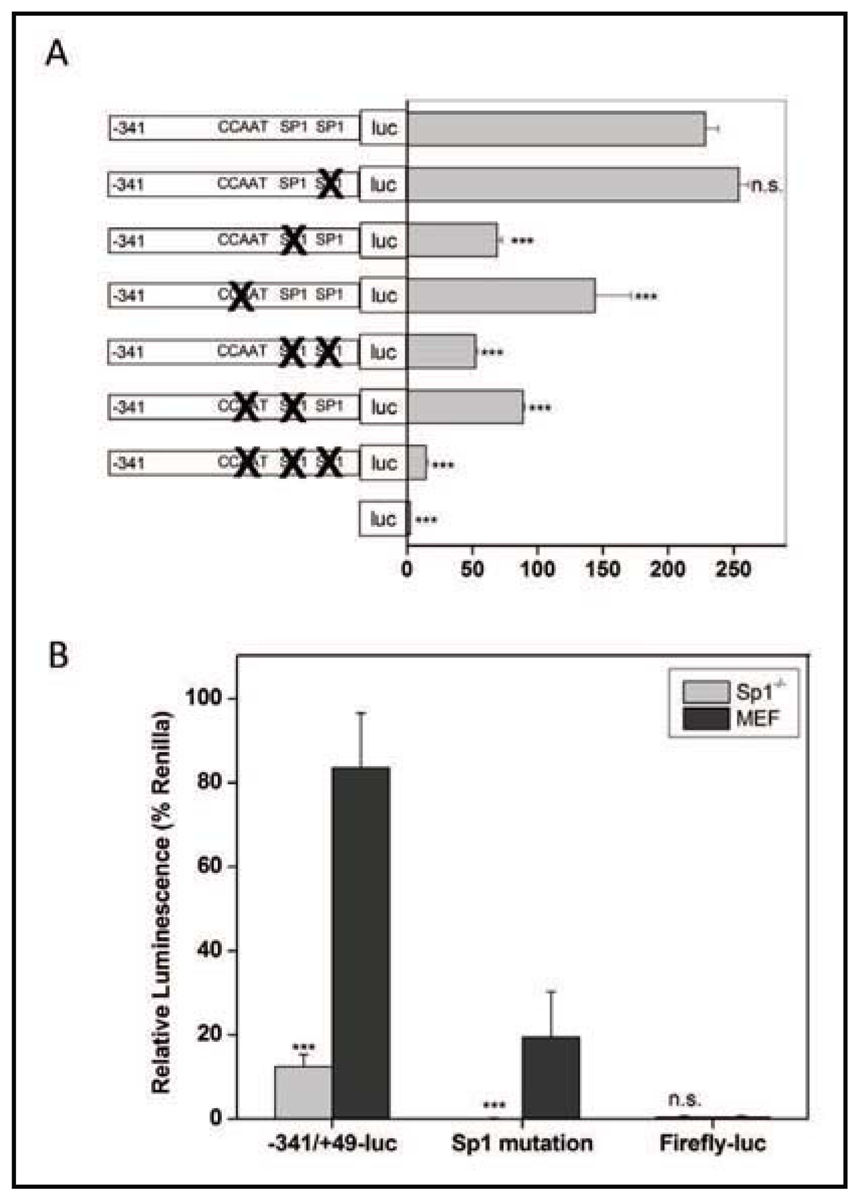
Mutational analysis of Sp1 and CCAAT binding regions of the *Slc38a3* promoter. A) The consensus sequences of Sp1 and the CCAAT motif were mutated in the −341/+49-luc construct. The single or combined mutations made are indicated by crosses. Significantly lower luminescence was observed when single or combined mutations of the CCAAT enhancer and the two Sp1 binding sites were created. Each bar represents the mean±s.d. of the luminescence from three dishes. One of three independent experiments is depicted. *** indicates *p*<0.0001, and n.s. indicates no significance for the student’s *t* test comparing the deletion constructs to the control (−341/+49-luc) construct). B) −341/+49-luc and −341/+49-luc with deletion of the Sp1 binding sites (Sp1 mutation) were expressed in MEF and *Sp1*^−/−^ cells. The promoter-less firefly-luc was used as a background control. Each bar represents the mean±s.d. of the luminescence from three dishes. One of three independent experiments is depicted. *** indicates *p*<0.0001, and n.s. indicates no significance for the student’s *t* test comparing each construct to −341/+49-luc expressed in MEF cells.

To examine whether the mouse *Slc38a3* gene could be transcriptionally up-regulated during acidosis, we analysed the mouse *Slc38a3* promoter using reporter gene constructs. The *Slc38a3* gene has a slightly unusual structure with the AUG translation initiation codon found in exon 2. An 8kb intron separates exon 1 and exon 2. Thus full length protein could be produced from a promoter in front of exon 1, but also by an alternative promoter in front of exon 2. To test the possibility of an alternative promoter located in front of exon 2, we performed reporter gene assays of ~1.9kb fragments located upstream of exon 1 and exon 2. The experiments revealed promoter activity only in front of exon 1, but not in front of exon 2 (Fig. 3A). Thus, all subsequent studies focused on the promoter adjacent to exon 1 spanning the region −1841/+49, relative to the transcription start site. To understand the mechanism of gene transcription, the −1841/+49-luc construct was transfected into four different cell lines (Fig. 3A). HepG2 cells are human hepatoma cells that express human SNAT3 mRNA and protein endogenously, whilst HEK293 (Human Embryonic Kidney cells), HeLa (Cervical cancer cells) and FRTC (Fischer Rat Thyroid Cells) do not express the gene (Fig. 3B). Interestingly, significant promoter activity was detected with the −1841/+49-luc construct in all four cell lines (Fig. 3A) and its intensity was not correlated with endogenous SNAT3 expression. The *Slc38a3* promoter thus appears to be active in many cell lines. To identify the core promoter, six deletions of the −1841/+49-luc construct were created spanning the regions from 1341, 841, 341, 161, 61 and 11bp upstream to 49bp downstream of the designated transcription start site. The relative luminescence of these constructs was measured in HEK293 cells (Fig 4A). Slight decreases in luminescence were observed with shortening of the upstream region. However, deletion of the region between −61 and −11 completely abolished promoter activity. Sequence analysis indicated the presence of two binding sites for the ubiquitous Sp1 transcription factor and one CCAAT enhancer motif in this region (Fig. 4B). These sequences were highly conserved across all vertebrates. To test this prediction, single or combined mutations of the Sp1 and CCAAT consensus sequences were created. Since the activity of the −341/+49-luc construct was consistently similar to that of the −1841/+49-luc construct (Fig. 4A), the mutations were created in the −341/+49-luc construct. Mutation of the Sp1 site closest to the transcription start site alone did not significantly reduce the promoter activity (Fig. 5A). Mutation of the second Sp1 site, by contrast, caused a significant reduction in promoter activity, which was further reduced by additional mutation of the first SP1 site. Moreover, mutation of the CCAAT enhancer alone or in combination with one or two Sp1 sites caused a significant reduction in the observed luminescence. Promoter activity was almost abolished when all three sites were mutated. Taken together, these results indicate that the *Slc38a3* core promoter lies merely 50bp upstream of the transcription start site where promoter activity is driven by the *cis* elements that bind Sp1 and factors binding to the CCAAT enhancer motif.

The expression of Sp1 in all tested cell lines (Fig. 3B) explained the ubiquitous activity of the promoter. This notion was confirmed by measuring the activity of the −341/+49-luc (wildtype) and −341/+49-luc (Sp1 and CCAAT mutated sites) in cells deficient in Sp1 (Fig. 5B). Sp1^−/−^ cells [30] and wild-type mouse embryonic fibroblasts (MEF) have previously been used to study Sp-1 mediated transcription [30]. Expression of the −341/+49-luc construct in Sp1^−/−^ cells had a significantly lower activity than that in MEF cells (Fig. 5B). Consistent with the previous findings, mutation of the Sp1 sites caused a significant reduction in promoter activity in MEF cells. Only background levels of luminescence were detected for this construct in Sp1^−/−^ cells.

**Fig. 6:**
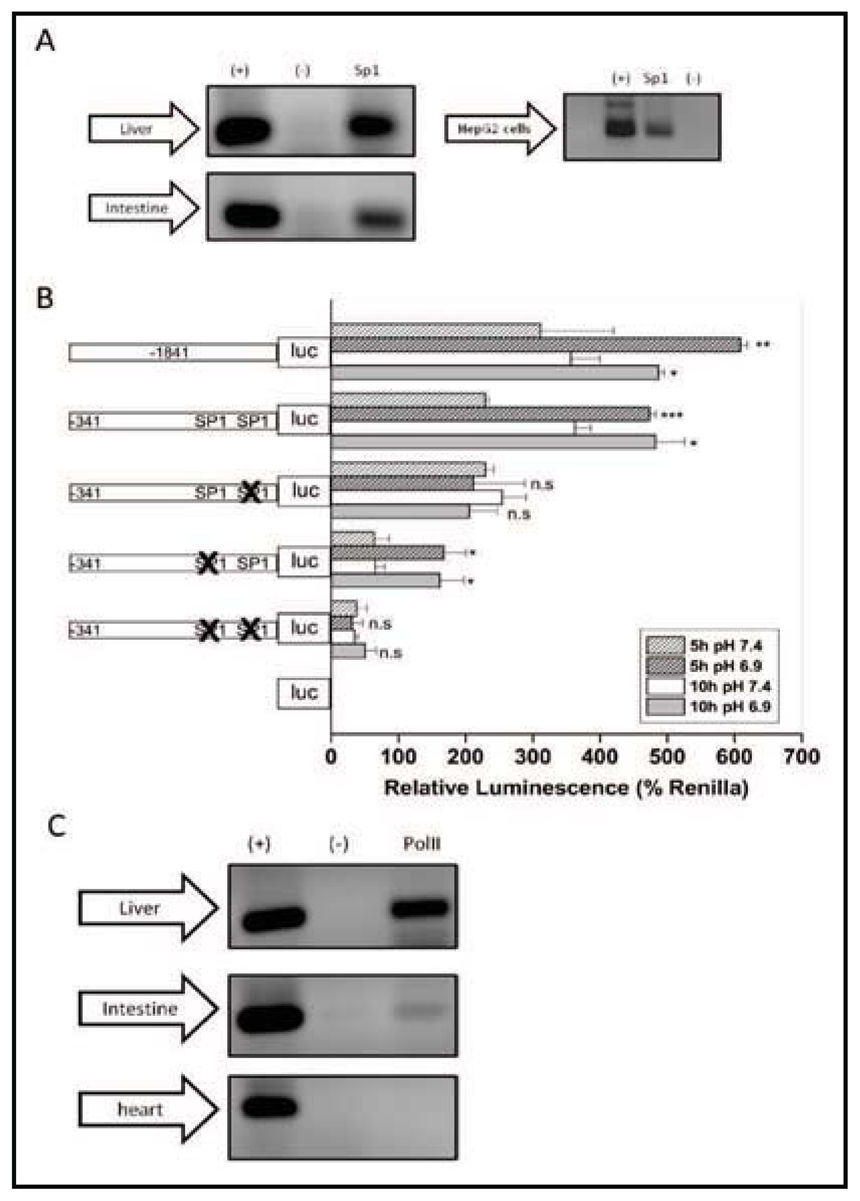
Binding of Sp1 to the *Slc38a3* promoter and its regulation by acidosis. A) Mouse liver and intestine, and HepG2 cells (as indicated) were cross-linked and extracted chromatin was subjected to immunoprecipitation with anti −IgG (−), histone H3 (+) or Sp1 antibodies. Purified DNA was subjected to PCR with *Slc38a3* promoter-specific primers. Immunoprecipitation with Sp1 shows a clear band at 150bp (arrow) indicating Sp1 binding to *Slc38a3 in vivo*. B) −1841/+49-luc, −341/+49-luc (wildtype or mutated Sp1 consensus sites – as indicated) were transfected into LLC-PK1F^+^ cells. The relative luminescence of the constructs was measured after 5h or 10h of incubation in media at pH 7.4 or 6.9. Each bar represents the mean±s.d. of the relative luminescence from three dishes. ***,** and * indicate *p*<0.0001, *p*<0.001 and *p*<0.01, respectively for student’s *t* test comparing the luminescence at pH 6.9 to pH 7.4. C) Proteins and DNA in mouse liver, intestine and heart were cross-linked and chromatin was immunoprecipitated with anti- IgG (−), histone H3 (+) or RNA Polymerase II (PolII) antibodies. Purified DNA was subjected to PCR with *Slc38a3-*specific primers. The 250bp *Slc38a3*-specific band is indicated by the arrow. While RNA Polymerase II bound to *Slc38a3* in the liver, no interactions were observed in the intestine or heart.

To test if the Sp1 transcription factor binds to the *Slc38a3* promoter *in vivo*, chromatin immunoprecipitation (ChIP) was performed on human HepG2 cells, mouse liver and mouse intestine. HepG2 cells and mouse liver express both, SNAT3 mRNA and protein [31], while intestine does not, although it does express Sp1. Immunoprecipitation of fragmented DNA with cross-linked proteins from mouse tissues and HepG2 cells was performed with anti-IgG (negative control), anti-Histone H3 (positive control) and anti-Sp1 antibodies. Subsequently PCR with *Slc38a3* promoter-specific primers was performed, resulting in the detection of Sp1 in all three samples (Fig. 6A). The IgG negative control (−) did not produce a detectable product, while the histone H3 antibody positive control (+) produced a strong band (Fig. 6A). This demonstrates an *in vivo* interaction between Sp1 and the *Slc38a3* promoter in mouse liver and intestine, and HepG2 cells.

Given that mRNA stability could not fully explain the up-regulation of mouse SNAT3 mRNA under acute or chronic acidotic conditions in primary cultures of renal cortical tubules, we wondered whether transcriptional activation was pH-sensitive. To this end *Slc38a3* reporter gene constructs −1841/+49-luc, −341/+49-luc and −341/+49-luc containing deletions of the Sp1 binding sites, were transfected into LLC-PK1-FBPase^+^ cells and subjected to 5h or 10h of acidosis at pH 6.9. As shown in Fig. 6B, a ~1.5–2-fold increase in the relative luminescence of −1841/+49-luc and −341/+49-luc constructs was observed after 5h acidosis, which declined during chronic acidosis (10h). As outlined above the *Slc38a3* core promoter contains two consensus sequences for Sp1 binding. Mutation of the Sp1 binding site closest to the transcription start-site did not affect basal promoter activity, but abolished the response to acidosis (Fig. 6B).

**Fig. 7:**
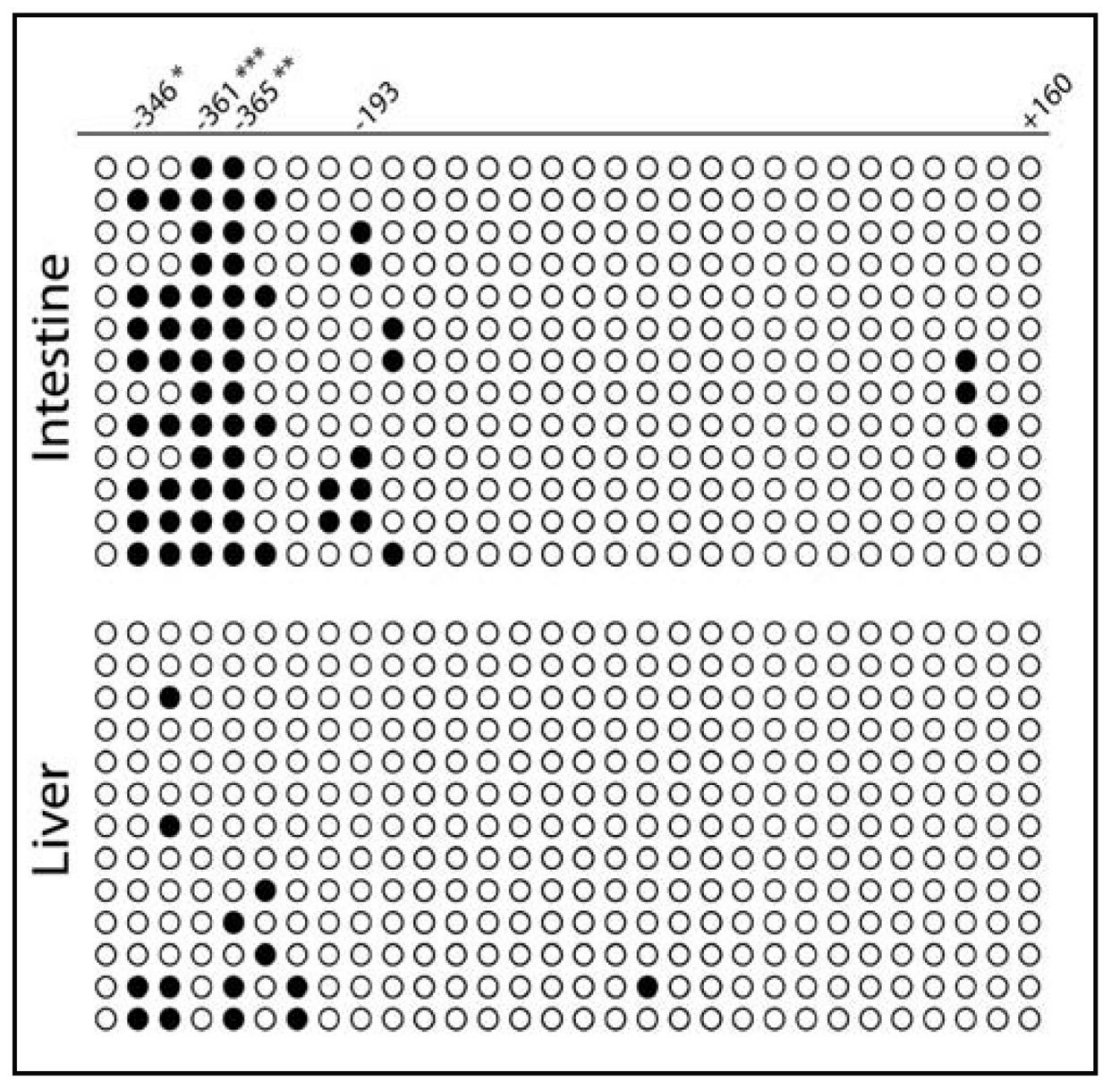
Differential methylation of *Slc38a3* in mouse intestine and liver. Genomic DNA from mouse intestine and liver were subjected to bisulfite conversion. *Slc38a3* DNA spanning −433 to −110 and −136 to +160 were amplified using nested PCR and cloned into the pCR-TOPO-XL vector and sequenced. Thirteen clones were analysed for each sample using the BioQAnalyzer program. The results indicate four CpG residues were differentially methylated in the intestine compared to the liver. Clear circles indicate unmethylated, whereas solid circles indicate methylated CpG residues. * indicates *p<0.01*, ** indicates *p*<0.001 and *** indicates *p*<0.0001 for the chi-squared test for the odds ratio comparing methylation levels between the liver and intestine.

The results thus far support a model in which acidosis increases both transcription from the Sp1 driven promoter and mRNA stability, particularly at early time points. In view of the ubiquitous distribution of Sp1, it would be expected that SNAT3 mRNA would be up-regulated under acidosis in many tissues. Using chromatin immunoprecipitation, we could indeed demonstrate binding of Sp1 to the *Slc38a3* promoter in the intestine, which does not express SNAT3 (Fig. 6A). However, in tissues that do not express SNAT3, such as intestine and heart, RNA polymerase II does not occupy the promoter and therefore transcription does not occur (Fig. 6C, see Discussion Fig. 9A for HepG2 cells). The lack of transcriptional activation in these tissues could be related to promoter methylation. Bisulfite genome sequencing of the *Slc38a3* promoter region from −433 to +160 relative to the transcriptional start site, revealed a number of differentially methylated residues. Four CpG residues positioned at −394, −365, −361, and −193 were found to be highly methylated in the intestine, but were barely methylated in liver (Fig. 7). While these residues are outside the core promoter they could interfere with the binding of factors that are required to initiate tissue-specific transcription or suppress expression in other tissues.

## Discussion

**Fig. 8:**
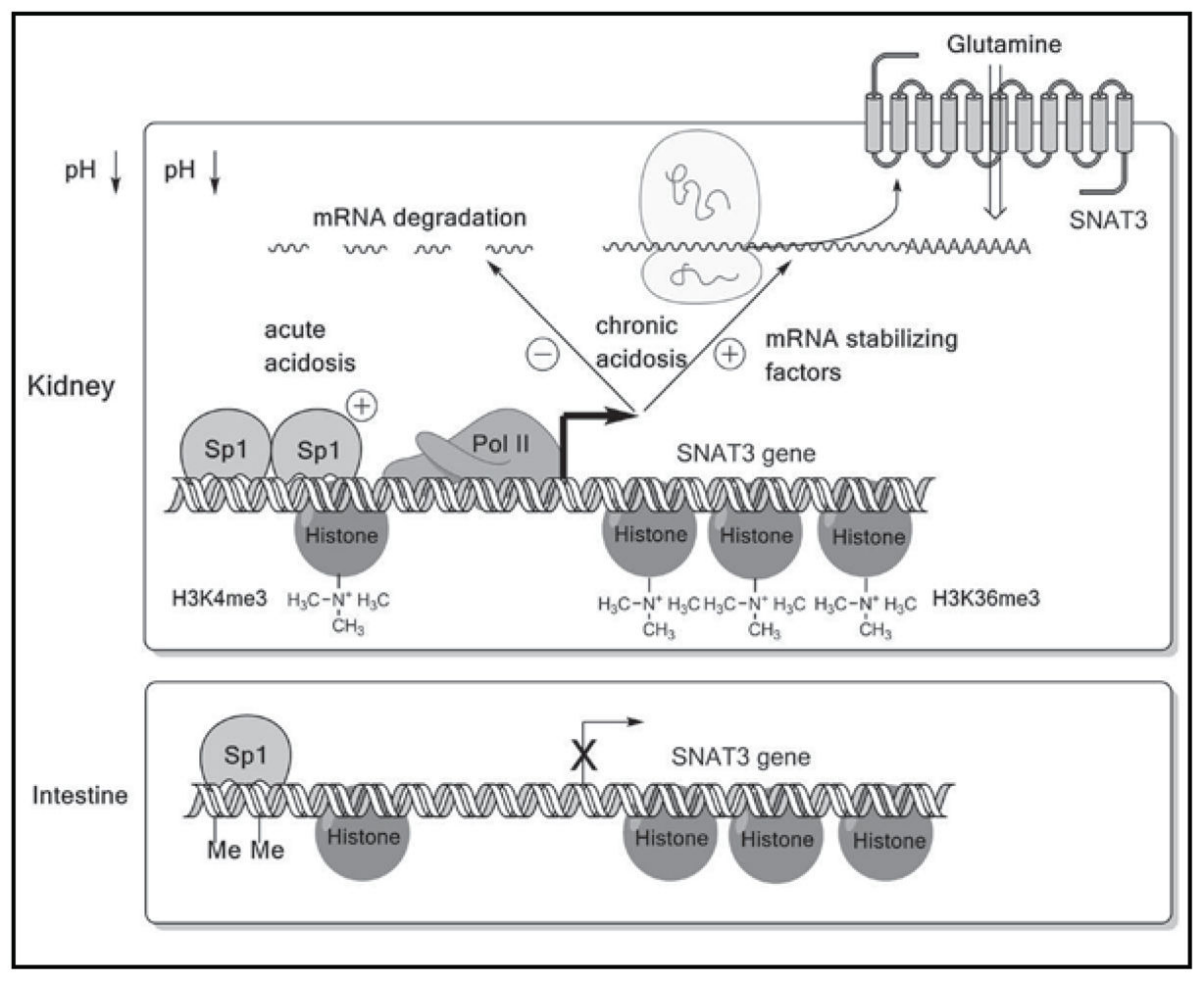
Overview of mechanisms regulating SNAT3 transcription and mRNA stability. Upper panel: Tubular epithelial cells in the kidney respond to acute acidosis by up-regulating *Slc38a3* transcription and to chronic acidosis by mRNA stabilisation. Histone signatures allow access of transcription factors to the promoter. Lower panel: Intestine as an example of a tissue which does not express *Slc38a3*. Promoter methylation and lack of an active histone signature are typical features of such tissues.

Acidosis causes a striking up-regulation of both SNAT3 mRNA and protein in the kidney [7–9]. To investigate molecular mechanisms that could contribute to this up-regulation we used LLC-PK1-FBPase^+^ cells, a well-established model of metabolic regulation in the proximal tubule. Our results suggest that during acute acidosis, up-regulation of SNAT3 mRNA is due to transcriptional activation, mainly triggered by enhanced binding of Sp1 to the *Slc38a3* promoter (Fig. 8). During chronic acidosis, by contrast, stabilisation of the SNAT3 mRNA becomes the dominant factor. Using the pβ-globin reporter mRNA, we could show that the SNAT3 3′UTR contains an AU-rich element that is responsible for destabilizing SNAT3-3′UTR-pBG at physiological pH. In view of the significant change in mRNA stability of the SNAT3-3′UTR-pBG construct, it was surprising to observe a very stable mRNA in primary cultures of mouse cortical tubules. However, primary cultures could express high levels of RNA stabilising factors that bind to AU-rich sequences such as AUF1 or HuR [26, 32], which would stabilise SNAT3 mRNA constitutively In addition primary cultures are grown in the presence of glucocorticoids. Glucocorticoids are released to protect the body against stress and hypoglycemia. Similar to acidosis, glucocorticoids up-regulate gluconeogenic enzymes and SNAT3. It has been shown that the response to acidosis is mediated in part by the release of glucocorticoids [33], which could explain the stable expression in primary cells. Provided that the Slc38a3 promoter does not contain a glucocorticoid response element [33], glucocorticoids may act through up-regulation of RNA stabilizing factors.

**Fig. 9:**
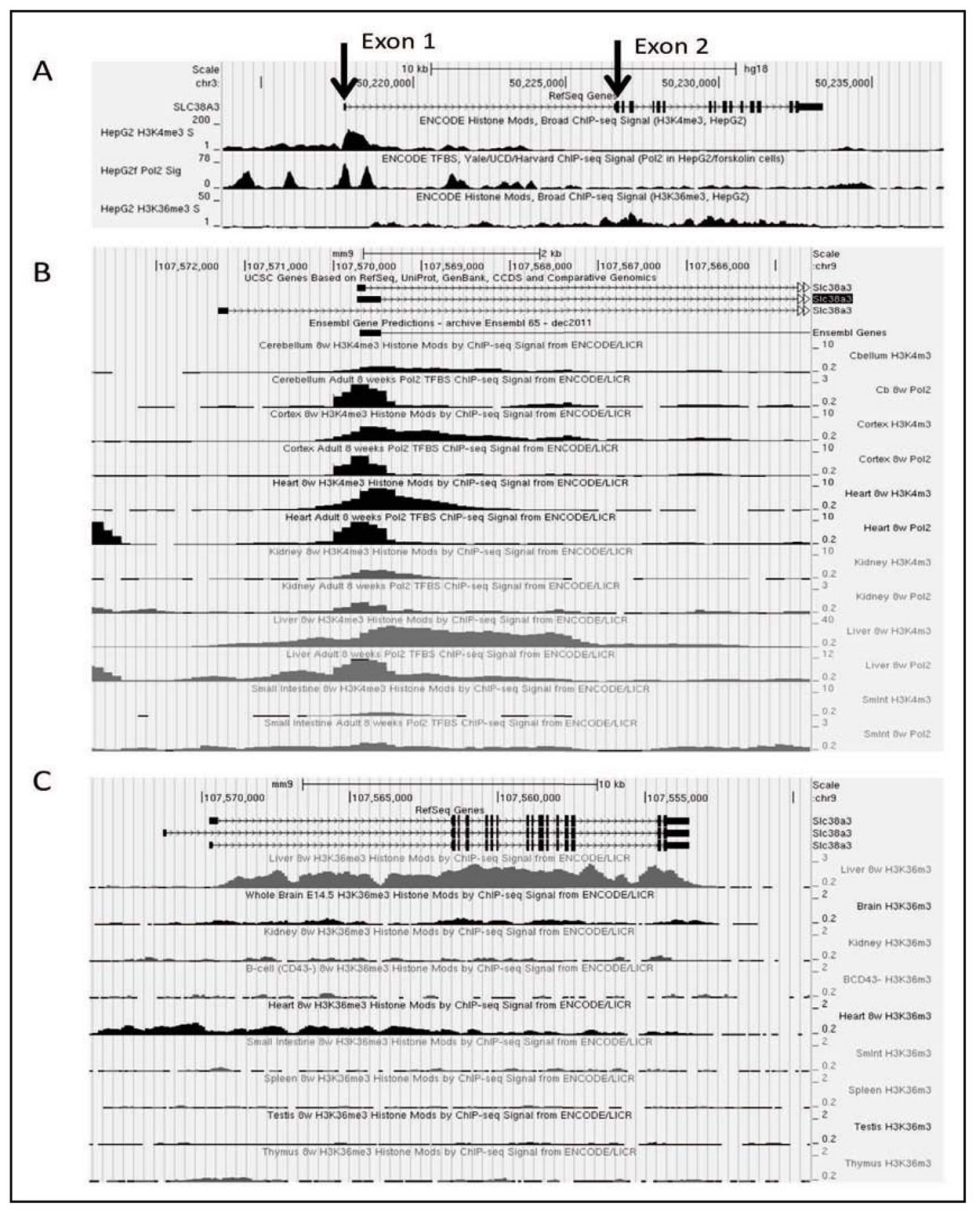
Histone modification and polymerase II binding to the *Slc38a3* promoter. A) The ENCODE database was used to extract histone modifications H3K4me3 and H3K36me3 and RNA polymerase II binding along the human Slc38a3 gene. HepG2 cells express SNAT3 endogenously (see Figure 2) and show an active promoter (high levels of H3K4me3) with a typical dip at the RNA polymerase 2 binding site just in front of Exon 1 (arrow), no such signature was observed upstream of exon 2 (arrow). The exons of SNAT3 show elevated levels of H3K36me3, typical for actively transcribed genes. B) The transcriptional start site of mouse *Slc38a3* was analysed through the ENCODE database for tissue-specific histone modification H3K4me3 (indicating open promoter) and RNA polymerase II binding. Tissues are Cerebellum (Cbellum or Cb), Brain cortex, heart, kidney, liver and small intestine (Smint) C) Presence of histone modification H3K36me3 (indicating actively transcribed genes) in different organs as analysed through the ENCODE database (BCD43-, B-cell CD43-; Smint, small intestine).

The *Slc38a3* gene appears to be transcribed from a promoter in front of the non-coding exon 1. Inspection of the ENCODE database confirmed this notion [34]. Histone variant H3K4Me3, indicating active promoter regions, was highly abundant over the DNA in front of exon 1 in HepG2 cells, which express SNAT3 endogenously (Fig. 9A). This site was also occupied by polymerase II. No such signature was observed in front of exon 2. To further support this notion, mRNAs with UTR’s arising upstream of exon 1 or exon 2 at normal and acidic pH could be detected using 5′RACE or RNase protection. Thus we cannot fully exclude the presence of a completely silent promoter, which would only be activated at acidic pH.

The pH-induced increase in transcriptional activity was abolished upon mutation of only one of the two Sp1-binding sites in the *Slc38a3* core promoter. Consistently, this Sp1-binding site had little influence on the basal transcriptional activity. It has previously been demonstrated that Sp1 is regulated by pH changes [35, 36]. Decreases in extracellular pH cause a concomitant decrease in the intracellular pH of proximal convoluted tubules and in LLC-PK1-FBPase^+^ cells [37, 38]. Low extracellular pH increases the DNA-binding capacity of Sp1, and further enhances its ability to interact with TATA-box binding protein to recruit the RNA Polymerase II complex [35]. In agreement with this notion, it has also been reported that higher levels of *Nhe3* mRNA during acute acidosis can be attributed to Sp1 dependent transcriptional up-regulation [39]. It is therefore possible that under normal conditions, Sp1 binds to the consensus sequence further upstream from the transcriptional start site. During acidosis, transcriptional synergy is generated by the binding of Sp1 to the second consensus sequence closest to the transcriptional start site causing an increase in *Slc38a3* transcription (Fig. 8). The Sp1 consensus sequence closest to the transcription start-site could therefore serve as a stress-induced transcription factor-binding site. It has been reported that SNAT3 expression decreases under acidotic conditions in glioma cells [40]. In contrast to LLC-PK1-FBPase^+^ cells, however, the intracellular pH of glioma cells was found to be more alkaline in acidotic media. Furthermore, proteins binding to the pH-RE in the SNAT3 mRNA may be different in both cell lines. For instance, the proposed regulator of glutaminase during acidosis, ζ-crystalline, is predominantly expressed in mouse and rat kidney, with little or no expression in liver and brain (Unigene database).

Under physiological conditions SNAT3 expression in the kidney cortex is almost absent, increasing dramatically during chronic acidosis. We therefore considered whether high expression in liver and brain were maintained by the same mechanism underlying high expression in kidney during acidosis. It appears, however, that epigenetic factors dominate tissue specific expression. In agreement with this notion, we found that tissues that express *Slc38a3* have de-methylated promoters and polymerase II occupies the transcriptional start site. This allows transcriptional activation by the ubiquitous Sp1 transcription factor. The ENCODE database [34] in addition shows enrichment of the active promoter indicating histone modification H3K4Me3 at the transcriptional start site of the Slc38a3 gene in HepG2 cells, liver, cerebellum and brain cortex (Fig. 9B, C). Surprisingly heart had high levels of H3K4Me3, as well, but not intestine. Accordingly, polymerase II was found occupying these promoters, while in the intestine the *Slc38a3* promoter neither showed H3K4Me3, nor polymerase II binding. Histone modification H3K36me3, which is found over actively transcribed genes was enriched in liver and to a lesser extent in brain (Fig. 9C). Heart showed some enrichment as well, but not over the exons, where it would be expected. Kidney also showed very little abundance of this modification suggesting that up-regulation during acidosis may include histone modification. The unique response of *Slc38a3* expression in the proximal tubule is most likely the combination of several factors: First, intracellular pH is sensitive to changes of the extracellular pH; second, a kidney-specific change of the histone signature, particularly H3K36me3 and third, kidney-specific expression of mRNA stabilizing proteins such as ζ-crystalline. SNAT3 expressing tissues that are not responsive to acidosis show constitutively stable expression of its mRNA, while tissues that do not express SNAT3, show promoter methylation and absence of an active histone signature (Fig. 8). While methylation was observed outside the core promoter, additional factors are required to explain lack of SNAT3 expression in cell lines, such as HEK, HeLa and FRTC that showed good reporter gene activity.

In summary SNAT3 expression is tightly regulated by transcription factor binding, mRNA stability and by epigenetic control mechanisms to mediate tissue-specific, cell-specific and pH-specific changes of mRNA levels, which then result in corresponding changes of protein abundance. It allows the SNAT3 transporter to carry out diverse roles such as neurotransmitter recycling, ammonia removal and gluconeogenesis in different cell types.

## Figures and Tables

**Table 1:** Primers used in this study. Incorporated restriction enzyme recognition sites are shown in bold, mutated triplets are underlined

Use	Species	Construct	Sense Primer (5′-3′)	Antisense primer (5′-3′)
cloning	Mouse	−1841/+49-luc	GCCTGTGTCTGAAATGTGGA	CGCTCGAGATCCGCTCTGCTCCAGGC
	Mouse	Promoter 2-luc	CGGTACCTAGGCTGCTCCGTATCCTG	CGCTAGCGGCTCAGAGACCACCCCA
	Mouse	−1341/+49-luc	GGAGTCTAATGGCCCTTCAC	GTACCGGCCAGTTAGGCCAG
	Mouse	−841/+49-luc	CAGCAGAGGCCATGGAATTA	GTACCGGCCAGTTAGGCCAG
	Mouse	−341/+49-luc	AAGCCTTCGGTATTCCTATT	GTACCGGCCAGTTAGGCCAG
	Mouse	−161/+49-luc	CCTATAGGCTTCGAGGTCAC	GTACCGGCCAGTTAGGCCAG
	Mouse	−61/+49-luc	CCTGGGATTGGCGGGCCGGG	GTACCGGCCAGTTAGGCCAG
	Mouse	−11/+49-luc	CATCCCCTGCTGCTGCAGTT	GTACCGGCCAGTTAGGCCAG
mutation	Mouse	−341/+49-luc	GGGGCGGGCCAGGGTTTGGGCCGGGCACCCTT	Reverse complement
	Mouse	−341/+49-luc	GATTGGCGGGCCGGGGTTTGGCCAGGGGCGGGGC	Reverse complement
	Mouse	−341/+49-luc	TCGGGCAGCCTGGGAAAAGCGGGCCGGGGGCGG	Reverse complement
RT-PCR	Mouse	*Slc38a3* Exon1	CTCAGCCTGGAGCAGAGC	ACGTTCAGCACACTCCGTTC
	Mouse	*Slc38a3* Exon2	TCTGAGCCATGGAGATACCC	AGAAGCCCCTCCAAGTGTTT
	Mouse	*Slc38a3* Exon3	AAACACTTGGAGGGGCTTCT	CCTCGAAATCGGTGAAGTGT
	Mouse/Rat/Human	*Slc38a3*	TTCTACAACGGGGTGGAGTC	ATCAGCAAGAAGCCAAGCAT
	Pig	*Slc38a3*	AGGGTGAGGGCTTCCTACAG	TGAAGAGGCTCTAGGGGACA
	Pig	*Slc38a3* 5′ *end of gene*	AGGGTGAGGGCTTCCTACAG	ATGATGGCGTTGCTAAGGTT
	Pig	*Slc38a3 3*′ *UTR*	AGTCATCCCCCTACCTTGCT	TGAAGAGGCTCTAGGGGACA
	Mouse/Rat/Human	*Sp*1	CCTCCATGCCAGGCCTCCAGA	TGTATGCCAGCGCAAGTGTG
	Mouse/Rat/Human	Actin	CATGGATGATGATATCGC	GGAGGAGCAATGATCTTGATCTTC
	Mouse/Rat/Human	*Gapdh*	GTCCATGCCATCACTGCCACCCAGA	GGATGACCTTGCCCACAGCCTT
	Mouse/Pig	18*S*	TTGACGGAAGGGCACCACCAG	GCACCACCACCCACGGAATCG
mRNA-stability	Mouse	*Slc38a3 3*′*UTR*	GCTCTAGAGCGATGACCTCCATC	GCTCTAGAGCTTGTGTATAATAT
	Mouse	*Slc38a3*	ACATCCCCTAGTCCTGCTGA	CCCTATGGAGGAGAGGGAAG
	pig	*Slc38a3*	AGTCATCCCCCTACCTTGCT	TGAAGAGGCTCTAGGGGACA
	Mouse	*Pepck*	TTTGAGATAGCGGCACAATG	GTGATTTCCCCTCCCAATCT
	Pig	*Pepck*	GCAAAGCACTTCTTCCCAAG	TCTTGGGCGAGATTACTGCT
ChIP	Mouse/Human	*Slc38a3 promoter*	GACCAGCCCCTCCTCCCGCG	TTCGCTCTGCTCCCGGCTGA
	Mouse	*Slc38a3 promoter*	AAACACTTGGAGGGGCTTCT	CCTCGAAATCGGTGAAGTGT
Bisulfitte	Mouse	−463 to +190	GAAGAAAAAAATTGATTGAGATAG	AACTTCCCAAAACCAACTATACAC
	Mouse	−433 to −110	TATTTATTTTTGTTAAGTAAAGGT	ACTTATCTCAAAAAAATAATC
	Mouse	−136 to+160	GATTATTTTTTTGAGATAAGT	AAATATTATAAACTCCTTCTA
